# A Prediction Equation to Assess Resting Energy Expenditure in Japanese Patients with COPD

**DOI:** 10.3390/jcm9113455

**Published:** 2020-10-27

**Authors:** Keisuke Morikawa, Kazuyuki Tabira, Hiroyuki Takemura, Shogo Inaba, Haruka Kusuki, Yu Hashitsume, Yuta Suzuki, Yosuke Tenpaku, Taro Yasuma, Corina N. D’Alessandro-Gabazza, Esteban Gabazza, Osamu Hataji

**Affiliations:** 1 Department of Rehabilitation, Matsusaka Municipal Hospital, Tonomachi 1550, Matsusaka, Mie 515-8544, Japan; morikawagacha@yahoo.co.jp (K.M.); pthiroyuki1988@gmail.com (H.T.); get_the_out_1209@yahoo.co.jp (S.I.); ah.jg.5296@outlook.jp (H.K.); hiromanayuukann@yahoo.co.jp (Y.H.); mchyu-ta@city-hosp.matsusaka.mie.jp (Y.S.); palu109291@gmail.com (Y.T.); 2 Department of Health of Science, Kio University Graduate School, Umamichu 4-2-2, Kitakatura-Gigunkoryocho, Nara 635-0832, Japan; k.tabira@kio.ac.jp; 3 Department of Immunology, Mie University Faculty and Graduate School of Medicine, Edobashi 2-174, Tsu, Mie 514-8507, Japan; t-yasuma0630@clin.medic.mie-u.ac.jp (T.Y.); dalessac@clin.medic.mie-u.ac.jp (C.N.D.-G.); 4 Respiratory Center, Matsusaka Municipal Hospital, Tonomachi 1550, Matsusaka, Mie 515-8544, Japan; mch1031@city-hosp.matsusaka.mie.jp

**Keywords:** COPD, resting energy expenditure, prediction equation, Japanese population, fat-free mass

## Abstract

Background: Medical nutrition therapy is important in the management of chronic obstructive pulmonary disease (COPD) patients. Determination of resting energy expenditure is essential to define therapeutic goals for medical nutrition. Previous studies proposed the use of equations to predict resting energy expenditure. No prediction equation is currently available for the Japanese population. The objective of this study was to develop an equation to predict resting energy expenditure in Japanese chronic obstructive pulmonary disease patients. To this end, we investigated clinical variables that correlate with the resting energy expenditure. Methods: This study included 102 COPD patients admitted at the Matsusaka Municipal Hospital Respiratory Center. We measured resting energy expenditure by indirect calorimetry and explored the relationship of resting energy expenditure with clinical variables by univariate and stepwise linear regression analysis. Results: The resting energy expenditure by indirect calorimetry was significantly correlated with fat-free mass, body weight, body mass index, height, gender, and pulmonary function test by univariate analysis. In the stepwise linear regression analysis, the fat-free mass, body weight, and age remained significantly correlated with indirect calorimetry’s resting energy expenditure. The fat-free mass, body weight, and age explained 50.5% of the resting energy expenditure variation. Conclusion: Fat-free mass, body weight, and age were significantly correlated with resting energy expenditure by stepwise linear regression analysis, and they were used to define a predictive equation for Japanese COPD patients.

## 1. Introduction

Chronic obstructive pulmonary disease (COPD) causes high mortality and morbidity worldwide [1]. There are approximately 5.3 million people over 40-years-old in Japan with a COPD diagnosis [2]. Malnutrition-associated underweight is a common observation and an independent prognostic factor in COPD patients [3,4]. Malnutrition in COPD has negative consequences on respiratory function, defense mechanisms against infection, and exercise tolerance [5]. Determination of resting energy expenditure (REE) is indispensable for appropriate support and management of nutritional status in COPD patients [6,7,8]. Indirect calorimetry can be used to calculate REE. However, determination by indirect calorimetry requires expensive equipment, which is available only in a limited number of clinical facilities [9]. Therefore, REE is generally estimated from the Harris–Benedict equation in clinical practice [10]. However, the Harris–Benedict equation has been created to calculate REE in healthy subjects between 21 and 70 years old. Consequently, this equation’s use may lead to underestimating or overestimating the real REE values in subjects with a disease or with a different age range [10]. COPD-specific prediction equations for REE have been previously reported by Moore et al. in the United States and by Nordenson et al. in Sweden [11,12]. However, there is currently no REE prediction equation for Japanese COPD patients. This study aimed to develop an equation to predict resting energy expenditure in Japanese COPD patients. To this end, we investigated clinical variables that correlate with the resting energy expenditure measured by indirect calorimetry.

## 2. Experimental Section

### 2.1. Patients

Patients hospitalized for COPD at Matsusaka Municipal Hospital Respiratory Center from September 2016 to August 2020 were enrolled in the study. The diagnostic criteria of COPD were based on guidelines of the 2017 Global Initiative for Chronic Obstructive Lung Disease (GOLD) [13]. Patients receiving oxygen therapy or lacking indirect calorimetry evaluation were excluded from the study (Table 1).

### 2.2. Ethical Statement

The Research Ethics Committee of Matsusaka Municipal Hospital approved (reception/approval No 180803-8-5) the protocol, and the investigation was performed following the Helsinki Declaration’s principles.

### 2.3. Anthropometry

The height of each patient was measured to the nearest 0.5 cm using a horizontal headboard. Body weight (B.W.) was measured using a digital scale. Body mass index (BMI) was calculated as weight in kilograms divided by the square of height in meters (kg/m^2^), and the fat-free mass (FFM) was measured by bioelectrical impedance analysis (BIA method) using an In-Body S10 (BIO-SPACE, Tokyo, Japan).

### 2.4. Laboratory Analysis

Blood for measuring serum albumin (ALB) and C-reactive protein CRP) was sampled in the early morning on the same day of indirect calorimetric analysis. Measurements were carried out at the Clinical Laboratory of Matsusaka Municipal Hospital.

### 2.5. Pulmonary Function Test

Parameters of lung function were measured using a spirometer (DISCOM-21 FXIII, CHEST Co., Tokyo, Japan).

### 2.6. Indirect Calorimetry

We used the computed open-circuit indirect calorimetry (Aero monitor AE100i, Minato Medical Science Co., Osaka, Japan) to measure the resting energy expenditure (icREE) [14]. Measurement was performed after overnight fasting and after resting for 30 min in the supine position. Appropriate flow and gas calibration was performed before each measurement. A pump flushed ambient air through the facemask at a constant rate. The respiratory exchange was performed continuously for 20 min after an equilibrium period of 10 min. Data of icREE were recorded every 30 s. The icREE was calculated from the oxygen consumption (VO2) and carbon dioxide production (VCO2) using the Weir equation as follows [15].

icREE = (3.94 × VO2 + 1.11 × VCO2) × 1.44

### 2.7. Predicted REE

The predicted REE (pREE) was calculated using the Harris–Benedict (pREE_HB_) and Moore (pREE_Moore_) equations as follows [10,11].

Male pREE_HB_ = 66.47 + 13.75 × W (body weight (kg)) + 5.0 × H (height (cm)) − 6.75 × A (age (year))

Female pREE_HB_ = 665.09 + 9.56 × W + 1.84 × H − 4.67 × A.

Male REE_Moore_ = 11.5 × W (body weight (kg)) + 952

Female REE_Moore_ = 14.1 × W(body weight (kg)) + 515

### 2.8. Statistical Analysis

The normal distribution of the data was assessed using the Shapiro–Wilk test. The relationship between two variables was evaluated by univariate analysis and the Pearson product–moment correlation. The relationship between multiple variables was evaluated by the stepwise linear regression analysis. Variables that correlate with icREE with a *p* < 0.15 were included in the stepwise linear regression analysis. The weakest correlated variables (*p* > 0.15) were removed during the stepwise analysis. Fixed bias was assessed by paired *t*-test. The presence of bias was defined as no 0 in 95% confidence interval between the icREE, pREE_HB_, pREE_Moore_, and the pREE calculated in this study (pREE_thistudy_). We considered that the icREE agrees with the pREE calculated using each predicted equation in the confidence interval between 90% and 110%, as reported in previous studies [16,17]. Statistical analysis was performed using SPSS Statistics version 25. *P* < 0.05 was considered as statistically significant.

## 3. Results

### 3.1. Relationship of icREE with Clinical Variables by Univariate Analysis

**Figure 1:**
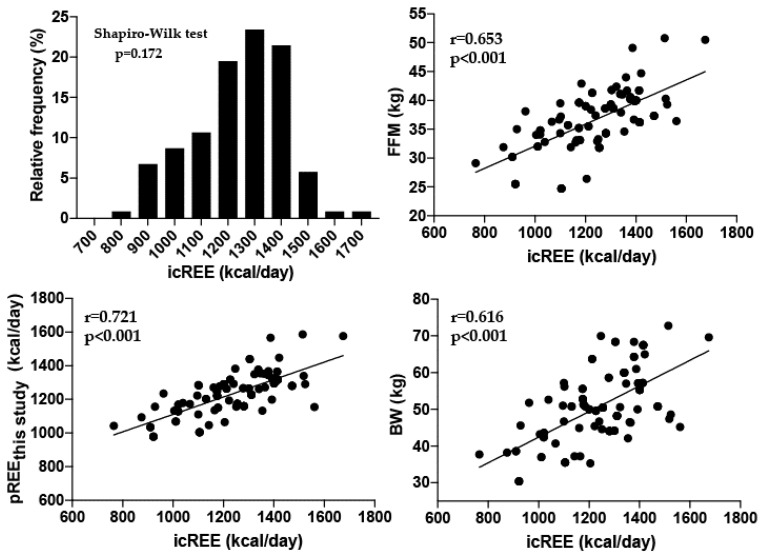
Distribution of icREE data and correlation of icREE with pREE, FFM, and BW. The Shapiro–Wilk test disclosed no significant difference (*p* = 0.172); therefore, the distribution was considered normal. The icREE was significantly correlated with pREE, FFM, and BW. icREE, resting energy expenditure measured by indirect expenditure; pREE_this study_, predicted resting energy expenditure calculated in this study; FFM, free-fat mass; BW, body weight.

This study included 102 patients and was conducted from September 2016 through August 2019. Table 1 describes the characteristics of the patients. According to the GOLD staging system, there were 9 COPD patients in stage I, 21 in stage II, 45 in stage III, and 27 in stage IV. Table 2 shows the strength of the relationship between icREE and other variables by univariate analysis. The icREE data showed a normal distribution based on the results of the Shapiro–Wilk test (Figure 1). The univariate analysis revealed that icREE is significantly correlated with FFM, body weight, BMI, height, %vital capacity, %forced vital capacity, and gender (Table 2, Figure 1). The icREE was not significantly correlated by univariate analysis with age, the ratio of forced expiratory volume in one second to forced vital capacity, %forced expiratory volume in one second, C-reactive protein, white blood cell count, or albumin (Table 2).

### 3.2. Relationship of icREE with Clinical Variables by Stepwise Linear Regression Analysis

Table 3 shows the strength of the relationship of icREE with variables by stepwise linear regression analysis. The factors that remained significantly correlated with the icREE during the stepwise linear regression analysis were fat-free mass, body weight, and age (Table 3). The remaining factors, including height, gender, pulmonary function parameters (%VC, %FVC, FEV_1_/FVC, %FEV_1_), C-reactive protein, white blood cell count, and albumin, showed no significant correlation with icREE in the stepwise regression analysis. Although the patients’ age was not significantly correlated with icREE by univariate analysis, it remained significantly related to icREE during the stepwise analysis (Table 3). FFM, body weight, and age explained 50.5% of variations observed in the icREE values. Therefore, we included these variables in the equation for predicted REE (pREE) as follows:

pREE_thisstudy_ (kcal/day) = 851.9 + 12.8 × FFM (kg) + 7.1 × BW (kg) − 5.7 × Age

### 3.3. Concordance Rate of icREE with Predicted REE

The values of pREE_HB_ (difference of 201.2 kcal; 95% coefficient interval between −227.2 and −175.1; *p* < 0.001) and pREE_Moore_ (difference of 271.0 kcal; 95% coefficient interval between 240.2 and 301.9); *p* < 0.001) significantly differed from the values of icREE, which were measured by indirect calorimetry. However, there was no significant difference between icREE and pREE_this study_ (difference of −4.5 kcal; 95% coefficient interval between 28.1 and 18.9; *p* = 0.7). The concordance rate of the icREE value with pREE_this study_ was 74.5%, with pREE_HB_ was 23.5%, and with pREE_Moore_ was 14.7% (Table 4).

## 4. Discussion

The objective of this study was to develop a formula to predict REE in Japanese patients with COPD. This study showed that icREE measured by indirect calorimetry is significantly and independently correlated with FFM, body weight, and age in Japanese patients with COPD. This study is the first to report an equation to predict REE in Japanese patients with COPD.

Consistent with previous studies, we showed here that FFM is the most potent influential factor of REE in COPD patients [12,18]. The present study revealed that FFM, together with body weight and age, explains approximately 51% of REE variations. Schols et al. reported that FFM explains about 34% of REE variations in patients with COPD and 84% in healthy elderly subjects, suggesting that besides FFM, other factors may also affect REE in COPD [19]. In the present study, we evaluated the contribution of many additional clinical variables, but their contribution to REE variations appeared to be minimum. In this connection, studies conducted in European populations showed a significant association of REE with the circulating level of systemic inflammatory markers, including C reactive protein and tumor necrosis factor-α, pointing to systemic inflammation as another potential confounding factor of REE in COPD [20,21]. We found no correlation of icREE with inflammatory indicators (CRP and WBC) in the present study. The patients’ different ethnic backgrounds may explain the discrepant results observed in the present study with previous reports. This observation suggests the need to develop a specific predicting equation for each ethnic population. Moreover, we found that the concordance rate of icREE with pREE_this study_ was high but very low with pREE_HB_ and pREE_Moore_, further supporting the accuracy of our equation model for the Japanese population.

## 5. Conclusions

This study showed a significant correlation between the resting energy expenditure measured by indirect calorimetry with the fat-free mass, body weight, and age by stepwise linear regression analysis. We used these three variables to define a predictive equation for Japanese COPD patients.

## Figures and Tables

**Table 1:** Patients characteristics.

Variables	All Patients	Staging I	Staging II	Staging III	Staging IV
Number of patients	102	9	21	45	
Age (year)	78.4 ± 6.3	78.7 ± 5.4	81.5 ± 5.7	79.1 ± 5.6	74.4 ± 6.8
Gender (M/F)	94/8	9/0	19/2	40/5	26/1
Height (cm)	159.0 ± 5.9	159.8 ± 3.5	159.2 ± 4.5	158.9 ± 7.2	158.7 ± 5.6
Body weight (kg)	51.7 ± 10.1	54.2 ± 8.0	53.5 ± 7.8	52.2 ± 10.8	44.3 ± 7.3
BMI (kg/m^2^)	20.4 ± 4.0	21.3 ± 3.5	21.1 ± 2.6	20.6 ± 4.0	17.5 ± 2.2
FFM (kg)	36.6 ± 5.1	37.6 ± 3.2	38.4 ± 3.3	36.5 ± 5.6	34.9 ± 5.5
VC (%pred)	75.5 ± 17.9	94.0 ± 18.9	87.3 ± 6.5	72.6 ± 15.1	62.9 ± 17.4
FVC (%pred)	74.3 ± 18.5	97.6 ± 18.3	86.4 ± 9.4	71.5 ± 16.2	60.6 ± 12.8
FEV₁/FVC (%pred)	47.6 ± 16.6	68.6 ± 6.7	57.6 ± 9.4	44.9 ± 13.3	29.8 ± 6.1
%FEV₁ (%pred)	46.7 ± 39.7	90.7 ± 5.7	64.3 ± 10.2	38.6 ± 5.5	24.3 ± 4.2
WBC (/μL)	7621 ± 2400	5742 ± 1340	7080 ± 1706	8549 ± 2548	7288 ± 2337
ALB (mg/dL)	3.3 ± 0.5	3.3 ± 0.7	3.0 ± 0.5	3.3 ± 0.5	3.4 ± 0.5
CRP (mg/dL)	0.73 ± 1.04	0.58 ± 0.57	0.92 ± 0.86	0.65 ± 0.66	0.79 ± 1.75
icREE (kcal)	1239 ± 173	1303 ± 207	1223 ±176	1248 ± 159	1205 ± 176

Data are the mean ± standard deviation (SD) of the mean. The clinical stages of the Global Initiative for Chronic Obstructive Lung Disease or GOLD are described. BMI, body mass index; FFM, free-fat mass; VC, vital capacity; FVC, forced vital capacity; FEV1, forced expiratory volume in 1-s percent; %FEV₁, percent forced expiratory volume in 1 s; WBC, white blood cell; ALB, serum albumin; CRP, C-reactive protein; icREE, resting energy expenditure measured by indirect calorimetry; pred, predicted.

**Table 2:** Relationship of resting energy expenditure measured by indirect calorimetry with clinical variables by univariate analysis.

	Correlation Coefficients	*p* Values
FFM (kg)	0.653	<0.001
Body weight (kg)	0.616	<0.001
BMI (kg/m^2^)	0.546	<0.001
Height (cm)	0.305	0.002
Gender (m/f)	–0.222	0.007
%VC (%predicted)	0.270	0.006
%FVC (%predicted)	0.223	0.024
FEV₁/FVC (%predicted)	0.150	0.131
%FEV₁ (%predicted)	0.138	0.165
Age (years)	−0.172	0.128
CRP (mg/dL	0.141	0.160
WBC (/μL)	0.114	0.253
ALB (mg/dL)	−0.026	0.797

BMI, body mass index; VC, vital capacity; FVC, forced vital capacity; FEV₁, forced expiratory volume one second; CRP, C reactive protein; %FEV₁, percent forced expiratory volume one second; WBC, white blood cell; ALB, serum albumin; m, male; f, female.

**Table 3:** Relationship of icREE with clinical variables by stepwise linear regression analysis.

**Model 1**				
Variable	SPRC	SPRC SE	β	*p*-value
Constant	423.1	95.5		<0.001
FFM	22.3	2.6	0.653	<0.001
**R2 = 0.421**				
**MODEL 2**				
Variable	SPRC	SPRC SE	β	*p*-value
Constant	409.2	91.4		<0.001
FFM	14.9	3.4	0.436	<0.001
BW	5.6	1.8	0.318	0.002
**R2 = 0.470**				
**MODEL 3**				
Variable	SPRC	SPRC SE	β	*p*-value
Constant	851.9	179.4		<0.001
FFM	12.8	3.3	0.376	<0.001
BW	7.1	1.8	0.404	<0.001
Age	−5.7	2.0	0.208	0.006
**R2 = 0.505**				

FFM, free fat mass; B.W., body weight; REE, resting energy expenditure; SPRC, standardized partial regression coefficient; S.E., standard error.

**Table 4:** The concordance rate between REE measured by indirect calorimetry. (icREE) and REE calculated by prediction equations (pREE).

	Mean	± SD	*p* Value	Concordance Rate (%) with icREE
icREE	1238 ± 173			
pREE_this study_	1234 ± 124	0.701	74.5	
pREE_HB_	1038 ± 135	*p* < 0.001	23.5	
pREE_Moore_	1510 ± 163	*p* < 0.001	14.7	

icREE, resting energy expenditure measured by indirect calorimetry; pREE, predicted resting energy expenditure; SD, standard deviation.

## References

[1] Diaz-Guzman E., Mannino D.M. (2014). Epidemiology and prevalence of chronic obstructive pulmonary disease. Clin. Chest. Med..

[2] Fukuchi Y., Nishimura M., Ichinose M., Adachi M., Nagai A., Kuriyama T., Takahashi K., Nishimura K., Ishioka S., Aizawa H. (2004). COPD in Japan: The Nippon COPD Epidemiology study. Respirology.

[3] Hallin R., Gudmundsson G., Suppli Ulrik C., Nieminen M.M., Gislason T., Lindberg E., Brondum E., Aine T., Bakke P., Janson C. (2007). Nutritional status and long-term mortality in hospitalised patients with chronic obstructive pulmonary disease (COPD). Respir. Med..

[4] King D.A., Cordova F., Scharf S.M. (2008). Nutritional aspects of chronic obstructive pulmonary disease. Proc. Am. Thorac. Soc..

[5] Schols A.M., Soeters P.B., Dingemans A.M., Mostert R., Frantzen P.J., Wouters E.F. (1993). Prevalence and characteristics of nutritional depletion in patients with stable COPD eligible for pulmonary rehabilitation. Am. Rev. Respir. Dis..

[6] Creutzberg E.C., Wouters E.F., Mostert R., Weling-Scheepers C.A., Schols A.M. (2003). Efficacy of nutritional supplementation therapy in depleted patients with chronic obstructive pulmonary disease. Nutrition.

[7] Sridhar M.K., Galloway A., Lean M.E., Banham S.W. (1994). An out-patient nutritional supplementation programme in COPD patients. Eur. Respir. J..

[8] Weekes C.E., Emery P.W., Elia M. (2009). Dietary counselling and food fortification in stable COPD: A randomised trial. Thorax.

[9] McDoniel S.O. (2007). Systematic review on use of a handheld indirect calorimeter to assess energy needs in adults and children. Int. J. Sport Nutr. Exerc. Metab..

[10] Harris J.A., Benedict F.G. (1918). A Biometric Study of Human Basal Metabolism. Proc. Natl. Acad. Sci. USA.

[11] Moore J.A., Angelillo V.A. (1988). Equations for the prediction of resting energy expenditure in chronic obstructive lung disease. Chest.

[12] Nordenson A., Gronberg A.M., Hulthen L., Larsson S., Slinde F. (2010). A validated disease specific prediction equation for resting metabolic rate in underweight patients with COPD. Int. J. Chron. Obstruct. Pulmon. Dis..

[13] Hogman M., Sulku J., Stallberg B., Janson C., Broms K., Hedenstrom H., Lisspers K., Malinovschi A.N. (2018). 2017 Global Initiative for Chronic Obstructive Lung Disease reclassifies half of COPD subjects to lower risk group. Int. J. Chron. Obstruct. Pulmon. Dis..

[14] Inoue M., Sasaki M., Takaoka A., Kurihara M., Iwakawa H., Bamba S., Ban H., Andoh A. (2015). Changes in energy metabolism after induction therapy in patients with severe or moderate ulcerative colitis. J. Clin. Biochem. Nutr..

[15] Weir J.B. (1949). New methods for calculating metabolic rate with special reference to protein metabolism. J. Physiol..

[16] Frankenfield D., Roth-Yousey L., Compher C. (2005). Comparison of predictive equations for resting metabolic rate in healthy nonobese and obese adults: A systematic review. J. Am. Diet. Assoc..

[17] Weijs P.J. (2008). Validity of predictive equations for resting energy expenditure in U.S. and Dutch overweight and obese class I and II adults aged 18–65 y. Am. J. Clin. Nutr..

[18] Creutzberg E.C., Schols A.M., Bothmer-Quaedvlieg F.C., Wouters E.F. (1998). Prevalence of an elevated resting energy expenditure in patients with chronic obstructive pulmonary disease in relation to body composition and lung function. Eur. J. Clin. Nutr..

[19] Schols A.M., Fredrix E.W., Soeters P.B., Westerterp K.R., Wouters E.F. (1991). Resting energy expenditure in patients with chronic obstructive pulmonary disease. Am. J. Clin. Nutr..

[20] Broekhuizen R., Wouters E.F., Creutzberg E.C., Schols A.M. (2006). Raised CRP levels mark metabolic and functional impairment in advanced COPD. Thorax.

[21] Nguyen L.T., Bedu M., Caillaud D., Beaufrere B., Beaujon G., Vasson M., Coudert J., Ritz P. (1999). Increased resting energy expenditure is related to plasma TNF-alpha concentration in stable COPD patients. Clin. Nutr..

